# Metal-Multilayer-Dielectric Structure for Enhancement of *s*- and *p*-Polarized Evanescent Waves

**DOI:** 10.1186/s11671-016-1274-3

**Published:** 2016-02-01

**Authors:** Svitlana G. Ilchenko, Ruslan A. Lymarenko, Victor B. Taranenko

**Affiliations:** International Center “Institute of Applied Optics”, National Academy of Science of Ukraine, Kyiv, Ukraine

**Keywords:** Surface plasmon resonance, Evanescent wave, Polarization holography

## Abstract

We propose a structure based on combination of multilayer stack of dielectric films and thin metal layer for excitation and enhancement of both *s*- and *p*-polarized evanescent waves. It is shown that two different mechanisms of evanescent wave excitation may occur at the same angle of light beam incidence on the structure. Application for evanescent wave polarization holographic recording with the help of this structure is discussed.

## Background

Evanescent waves are waves that propagate along the boundary between two media and penetrate in them over a distance less than the wavelength. In such waves, all the energy concentrates in a narrow area near the boundary. That is why evanescent waves are very attractive for optical characterization with subwavelength resolution. The evanescent waves can be excited by various methods: diffraction on edge, total internal reflection, surface plasmons, multilayer dielectric structures, etc. The first two methods do not provide fields of high intensity on the surface. To obtain high-intensity electromagnetic field (high compared to the inner-atom field) and not to destroy the sample, one can use methods of the local field enhancement.

The enhanced field can be obtained in multilayer dielectric structure for *s*-polarized light [1]. This structure consists of *N* series of high *n*_H_ and low *n*_L_ refractive index quarter wavelength dielectric layers. Besides, the final layer should have low refractive index, and it is chosen to maximize the intensity of the evanescent wave. It was shown that enhancement factor can be derived from the standard method [2] for describing the propagation of light in dielectric layers and looks as follows:

1 $$ \frac{T}{T_0}=\frac{Y_{\mathrm{H}}^{2\mathrm{N}+2}\left({n}_{\mathrm{S}}^2-1\right)}{Y_{\mathrm{L}}^{2\mathrm{N}}{Y}_{\mathrm{S}}^2\left({n}_{\mathrm{L}}^2-1\right)}, $$

where *T* is transmitted intensity, *T*_0_ is transmission without coating, and *Y*_*i*_ = *n*_*i*_ ⋅ cos(*θ*_*i*_), where *i* denotes the corresponding layers: high (H), low (L), or substrate (S). It demonstrated experimentally the enchantment factor about 77 [1].

One of the popular methods of local field enhancement is surface plasmon resonance (SPR) excitation by *p*-polarized light. SPR occurs when the wave vector of surface plasmon is equal to the parallel wave vector of the evanescent wave by the total internal reflection (TIR) conditions on the prism-medium boundary [3]:

2 $$ \frac{2\pi }{\lambda}\sqrt{\frac{n_{\mathrm{H}}^2\cdot {n}_M^2}{n_{\mathrm{H}}^2+{n}_M^2}}=\frac{2\pi }{\lambda }{n}_{\mathrm{S}} \sin \left({\theta}_{\mathrm{S}\mathrm{PR}}\right), $$

where index *M* corresponds to the metal layer with complex refractive index [4]. Equation 2 defines the angle of SPR *θ*_SPR_ in the Kretschmann configuration. The dielectric layer can be added to control the dispersive curve [5] or the air gap can be placed between two metal layers to store the SPR evanescent waves in the planar microcavity [6].

## Presentation of the Hypothesis

Is it possible to enhance evanescent waves in both polarizations simultaneously at the same structure? Our idea is to combine the dielectric multilayer structure (DMS) and SPR methods for exciting both *s*- and *p*-polarized evanescent waves. We suppose that DMS can preserve the enhancement of the *s*-polarized evanescent wave after a thin metal layer adding. At the same time, SPR should provide a proper enhancement for *p*-polarized evanescent wave.

**Fig. 1:**
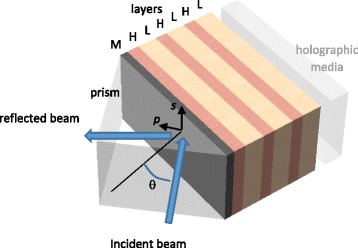
The scheme of metal (*M*)-multilayer-dielectric (stack of high (*H*) and low (*L*) refractive index layers) structure for optimization of enhanced evanescent wave at both polarizations

The proposed structure based on the combination of quasi quarter wave HL dielectric layers and a thin metal layer is shown in Fig. 1.

## Testing the Hypothesis

To obtain the electric **E** and magnetic **H** fields produced by the propagation of plane wave in a multilayer structure, we solve the Helmholtz in Eq. 3 within each layer with appropriate boundary conditions [7]

3 $$ \left({\nabla}^2+{k}^2\right)\left\{\begin{array}{c}\hfill \mathbf{E}\hfill \\ {}\hfill \mathbf{H}\hfill \end{array}\right\}=0, $$

where ∇^2^ is the transverse Laplasian and *k* is the wavenumber. We use the implementation of the *S*-matrix algorithm [8] that prevents numerical instabilities by taking into account a slightly absorbing permittivity and permeability of the dielectric layers. It is important because of complex refractive index of thin metal layer and high value of electromagnetic field in the structure.

We consider the DMS layers combined with complex refractive index metal layer [4] (relative permittivity for Ag is *ε* = −9.2931 + *i*0.87201 at 532-nm wavelength) with thickness of 50 nm. The layer of low refractive index is SiO_2_ (*n* = 1.4607) and the layer of high index is TiO_2_ (*n* = 2.6678). The prism, as we consider in our calculations, has refractive index of 1.52.

The optimization of layer thickness is started from the sequence of quarter-wave HL structure consisting of six dielectric layers. A deviation from quarter-wave stack of dielectric layers is widely used to achieve desirable dispersion curve that causes very significant enhancement of the internal electric field [9]. The numerical modeling shows that the desirable effect is present for quasi-periodical structures with thickness greater that quarter wavelength, namely: 1.54 *λ*/4 for the first H-layer, 1.4 *λ*/4 for the next H-layers, *λ*/4 for all L-layers except the last L-layer which is 1.866 *λ*/4.

**Fig. 2:**
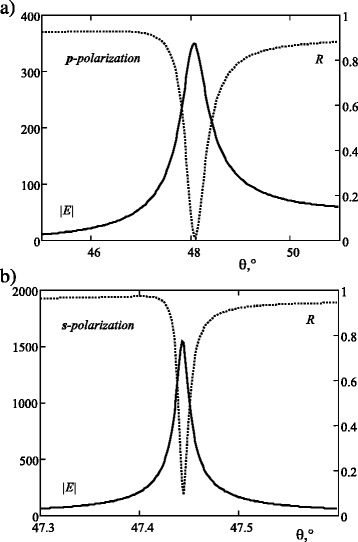
Enhancement factor (*solid line*) on interface “structure-air” and reflectance (*dashed line*) vs incident angle *θ* for **a** *p*- and **b** *s*-polarized incident light

The calculation shows that the angular resonances for *s*- and *p*-polarizations can overlap, and the intensity dip in reflectance at both polarizations occurs. The structure is non-transmitting TIR at the considered angle of incidence. The dependencies of electric field |E| on interface “structure-air” on the incident angle *θ* for *p*- and *s*-polarized incident light are shown in Fig. 2. The factor of enhancement at the resonance angle is about 300 for *p*-polarized wave and about 1300 for *s*-polarization. The theoretical limit of enhancement factor for DMS is greater than 10,000 at the expense of extremely narrow acceptance angle. Figure 2b shows that the width of resonance is about 0.02°, which is comparable with laser beam divergence. It is worthy to note that the resonance of DMS is extremely sensitive to the imperfections of the last interface. This causes the difficulty of the structure manufacturing.

Summarizing, we show that two different mechanisms of evanescent wave excitation and enhancement (SPR and DMS resonance) can be realized at one structure simultaneously. Evanescent wave produced by SPR is transferred via DMS to the last interface, L-layer—air. At the same time, the metal layer does not prevent the significant enhancement of the internal electric field in DMS.

## Implications of the Hypothesis

It is shown that the proposed metal DMS allows excitation of both *s*- and *p*-polarized evanescent waves. It is possible to equalize the intensities and incident angle of the orthogonal polarizations by adjusting the thickness of dielectric and metal layers. The *s*- and *p*-polarization evanescent waves can be applied for holographic recording in polarization-sensitive media like bacteriorhodopsin [10] and liquid crystalline polymers [11–13]. Also, this structure can be used for many practical applications similar to [14–16].

Another question: where the holographic media should be placed? It is evident that we can change the last L-layer by photosensitive medium. The internal electric field at the last L-layer is higher or compatible with the evanescent wave outside of the structure.

## Abbreviations

DMS: dielectric multilayer structure
SPR: surface plasmon resonance
TIR: total internal reflection

## References

[1] Nesnidal RC, Walker TG (1996). Multilayer dielectric structure for enhancement of evanescent waves. Appl Opt.

[2] Knittl Z (1976). Optics of thin films.

[3] Tang Y, Zeng X, Liang J (2010). Surface plasmon resonance: an introduction to a surface spectroscopy technique. J Chem Educ.

[4] Polyanskiy MN. Refractive index database. Available at http://refractiveindex.info/?shelf=main&book=Ag&page=Rakic.

[5] Ozaki M, Kato J-i, Kawata S (2013). Color selectivity of surface-plasmon holograms illuminated with white light. Appl Opt.

[6] Wakamatsu T (2010). Characteristics of metal enhanced evanescent-wave microcavities. Sensors.

[7] Hansen WN (1968). Electric fields produced by the propagation of plane coherent electromagnetic radiation in a stratified medium. JOSA.

[8] Yuffa AJ, Scales JA (2012). Object-oriented electrodynamics S-matrix code with modern applications. J Comput Phys.

[9] Razskazovskaya O, Luu TT, Trubetskov M, Goulielmakis E, Pervak V (2015). Nonlinear absorbance in dielectric multilayers. Optica.

[10] Bazhenov VY, Soskin MS, Taranenko VB, Vasnetsov MV, Arsenault H (1989). Biopolymers for real-time optical processing. Optical processing and computing.

[11] Platé NA (ed) (1993) Liquid-Crystal Polymers. Springer Science + Business Media LLC. doi: 10.1007/978-1-4899-1103-2.

[12] Kozlovsky M, Lymarenko R, Wang L, Haase W. Chiral photochromic liquid crystalline polymers for holography applications. Proc. SPIE 5521, Organic Holographic Materials and Applications II, (26 October 2004); doi:10.1117/12.561452.

[13] Berezhniy EO, Burykin MM, Ilchenko SG, Ostroukh AP, Lymarenko RA. Dynamic holographic grating in liquid crystalline polymer, 6th International Conference on Advanced Optoelectronics and Lasers CAOL’ 2013, Conference Proceedings, 363

[14] Hartman P, Škereň M. Numerical analysis of color holograms based on surface-plasmons. Proc. SPIE 9442, Optics and Measurement Conference 2014, 94420B (7 January 2015).

[15] Deepthi S (2015). Surface plasmon holography for security application. International Journal of Advanced Research Trends in Engineering and Technology.

[16] Zhang M, Meng L, Chun G, Cunningham BT (2014). Plasmonic external cavity laser refractometric sensor. Opt Express.

